# Optimising total knee replacement imaging: a novel 3D printed PET/CT anthropomorphic phantom for metal artefact simulation

**DOI:** 10.1186/s40658-024-00634-2

**Published:** 2024-03-28

**Authors:** Rajeh Assiri, Karen Knapp, Jon Fulford, Junning Chen

**Affiliations:** 1 Department of Radiological Sciences, College of Applied Medical Sciences, King Khalid University, 61421 Abha, Saudi Arabia; 2 Department of Medical Imaging, Faculty of Health and Life Sciences, The University of Exeter, South Cloisters, University of Exeter, St Luke’s Campus, Heavitree Road, Exeter, EX1 2LU UK; 3 College of Engineering, Mathematics and Physical Sciences, The University of Exeter, Exeter, UK

**Keywords:** PET/CT, Total knee replacement, TKR, 3D printing, Phantom, Bone

## Abstract

**Purpose:**

Arthroplasty phantoms, including total knee replacement (TKR) phantoms, have been frequently used to test metal artefact reduction methods applied to positron emission tomography/computed tomography (PET/CT) images. These phantoms generally simulate either simple anatomical features or simple activity distribution around the metal inserts in the PET/CT scans. 3D printing has been used recently to fabricate fillable anthropomorphic phantoms that accurately simulate volume and geometry. This study aims to describe the process of image segmentation, phantom modelling, 3D printing and validation of a population-based fillable TKR phantom that simulates human TKR PET/CT metal artefacts.

**Methods:**

10 participants (5 male and 5 female) were scanned using 3T MRI and the images were segmented to create average male and average female 3D knee models, inversely with void cortical and porous trabecular compartments for 3D printing and contrast media. Virtual total knee replacement (TKR) surgery was implemented on these models to prepare the insertion locations for knee prosthetic implants. Subsequently, TKR models were printed using a 3D photopolymer resin printer and then injected with normal saline to test the phantoms for any leaks. Subsequently, diluted iodinated contrast media was injected into the cortical compartment and saline with ^18^F-FDG was injected into the trabecular compartment and the phantom was scanned with PET/CT. The images were then evaluated and compared to the human knee radiographic features reported in the literature.

**Results:**

Phantoms were shown to be fluid-tight with distinct compartments. They showed comparable volume and geometry to the segmented human MRI knees. The phantoms demonstrated similar values for x-ray attenuation and Hounsfield units (HU) to the literature for both cortical and trabecular compartments. The phantoms displayed a uniform distribution for the radioactive tracer, resembling that seen in human trabecular bone PET. TKR phantom PET/CT images with metal inserts replicated the clinical metal artefacts seen clinically in the periprosthetic area.

**Conclusion:**

This novel, 3D-printed, and customisable phantom effectively mimics the geometric, radiographic and radiotracer distribution features of real TKRs. Importantly, it simulates TKR image metal artefacts, making it suitable for repeatable and comprehensive evaluation of various metal artefact reduction methods in future research.

## Introduction

PET/CT is a valuable diagnostic tool that provides morphological and functional information on oncological and non-oncological musculoskeletal diseases using either fluorine-18 fluorodeoxyglucose (^18^F-FDG) or ^18^F-sodium fluoride (^18^F-NaF) [1]. Moreover, PET/CT helps to monitor patients after joint arthroplasties such as total knee replacement (TKR) to characterise bone mineralisation around the prosthesis, detect early signs of aseptic loosening, differentiate between septic and aseptic prosthetic loosening, and assess the effectiveness of bone-active treatments in patients with reduced bone mineral density [2–4]. While PET/CT imaging has proven to be highly valuable in assessing TKR, the presence of metal artefacts in the CT images remains a significant challenge to obtaining clear and accurate images. Artefacts arise which can obscure the anatomical details in the CT images and impact on attenuation correction maps used for TKR PET scans [5].

Developing methods to address the issues associated with metal artefacts using human clinical data can be challenging due to ethical and practical constraints. Instead, using phantoms provides a valuable alternative approach. Phantoms allow for repeated scans and offer ground truth images that can be compared with optimised ones, making them a useful tool for developing and validating methods to minimize metal artefacts in clinical settings without compromising patient safety or ethical considerations. Phantoms composed of human tissue-mimicking materials have been widely used for in-vitro imaging to simulate clinical cases in pre-surgical or pre-radiotherapy planning [6]. A good anthropomorphic X-ray phantom should reflect human organ properties including geometry, density, attenuation coefficients, and Hounsfield Units (HU) if it is to be used for computed tomography (CT). Moreover, dedicated nuclear medicine and positron emission tomography phantoms must be injectable, mimicking the spatial distribution of the radiotracer found in the equivalent human tissue, and be stable and completely sealed to avoid any activity leaks [7]. Development of such a phantom that simulates both CT and PET required features is challenging and to the best of our knowledge, there is neither a patient-specific nor population-based customisable TKR phantom presented in the literature that contains all of these aforementioned features.

Several solid and liquid materials including metal-infused plastic filaments, Gypsum, and iodine have been used to mimic cortical bone in studies as they have a high atomic number and show comparable HU to bone [6, 8–11]. Metal-infused plastic filaments such as Bronzefill, Bismuth, and Copperfill however cause image artefacts when scanned with CT which affect the quantification of the activity levels in the artefact regions [11]. Moreover, for a phantom manufactured with Metal-infused plastic filaments, increasing the wall thickness leads to increased shielding of the contained radioactivity such that the phantom will effectively become like a shielded vial and give lower emission data than real bone [12]. Gypsum can be injected with ^18^F-NaF as it absorbs aqueous solutions; however, if a small amount of activity is injected in a large gypsum phantom, its diffusion will be uneven, and a hot spot of activity appears in the injection area [13]. Alternatively, fluids with similar HU to bone such as Iodinated contrast media when injected in any liquid-based phantom simulating bone will lead to an even distribution of the radioactivity, leading to more representative activity quantification [14].

3D printing has been used recently to produce state-of-the-art anthropomorphic phantoms for medical imaging and can generate high-resolution phantoms which can be hollow to allow the injection of radioactivity for nuclear medicine scans [7]. These phantoms can mimic the geometry, attenuation coefficient, and HU of human organs, depending on the chosen materials. Phantoms can either represent patient-specific or population-based models, generated from medical images or by using computer-aided design software (CAD) for virtually tailored scenarios. The model can be printed using different 3D printing technologies such as Stereolithography (SLA) and Fused deposition modelling (FDM) that can be applied using consumer-grade desktop printers [15]. In both SLA and FDM, the 3D model is sliced and converted into a stack of 2D layers, which are printed layer by layer. SLA uses a specific light wavelength to photopolymerize and cure liquid resin layer by layer to produce a solid 3D object while in FDM, the thermoplastic filament is melted in a moving extruder that builds the layers of the 3D object consecutively [15]. Both SLA and FDM printers are inexpensive and easy to operate but SLA-based printers such as Masked LCD stereolithography MSLA yield higher resolution prints than those printed with FDM [7, 15].

This study aims to establish a framework for developing separate population-based male and female PET/CT phantoms to be used with TKR implants such that TKR image metal artefacts can be studied, by combining medical imaging, CAD and modelling, injectable design, and 3D printing. To achieve this, magnetic resonance imaging (MRI) images of intact knees of human participants will be acquired and segmented to capture the morphology such that separate cortical and trabecular compartments of the phantom can be modelled. These compartments will be made injectable and fillable using CAD, to reflect the different attenuation features and distribution of the radioactive tracer, ^18^F-NaF as it is a promising radiotracer for distinguishing TKR complications with high sensitivity and specificity [16]. Virtual surgery will then be implemented on the models such that an insertion site for the knee implants will be generated. The models will then be printed with resin and scanned with PET/CT, and the radiographic features including HU and activity distribution assessed.

## Materials and methods

### Magnetic resonance imaging of healthy/intact knees

Ethical approval (eEMPS000388) was gained from the local research ethics committee at the University of Exeter to recruit healthy adult participants with no history of knee joint injury (5 males and 5 females) for knee joint magnetic resonance imaging (MRI). The average (± standard deviation) height, weight, and body mass index for the participants were 1.7 m (± 0.07), 76.8 kg (± 22.20) and 26.4 kg/m2 (± 6.20) respectively. Informed consent was obtained from all individual participants in the study. The knee joints of the participants were scanned in a supine position with a knee coil using a 3 Tesla MRI (Siemens, Prisma) to acquire T1 weighted images with a contrast that differentiated between soft tissue, cortical bone, and trabecular bone (see Table 1 for scanning protocol).

**Table 1:** MRI scanning protocol used for knee scans

FoV read	300 mm
FoV phase	72.2%
Slice thickness	1.0 mm
TR	11.70 ms
TE	3.48 ms
Flip angle	10^0^
In-plane resolution	1.04 × 1.04 mm

### Image segmentation and modelling

**Fig. 1:**
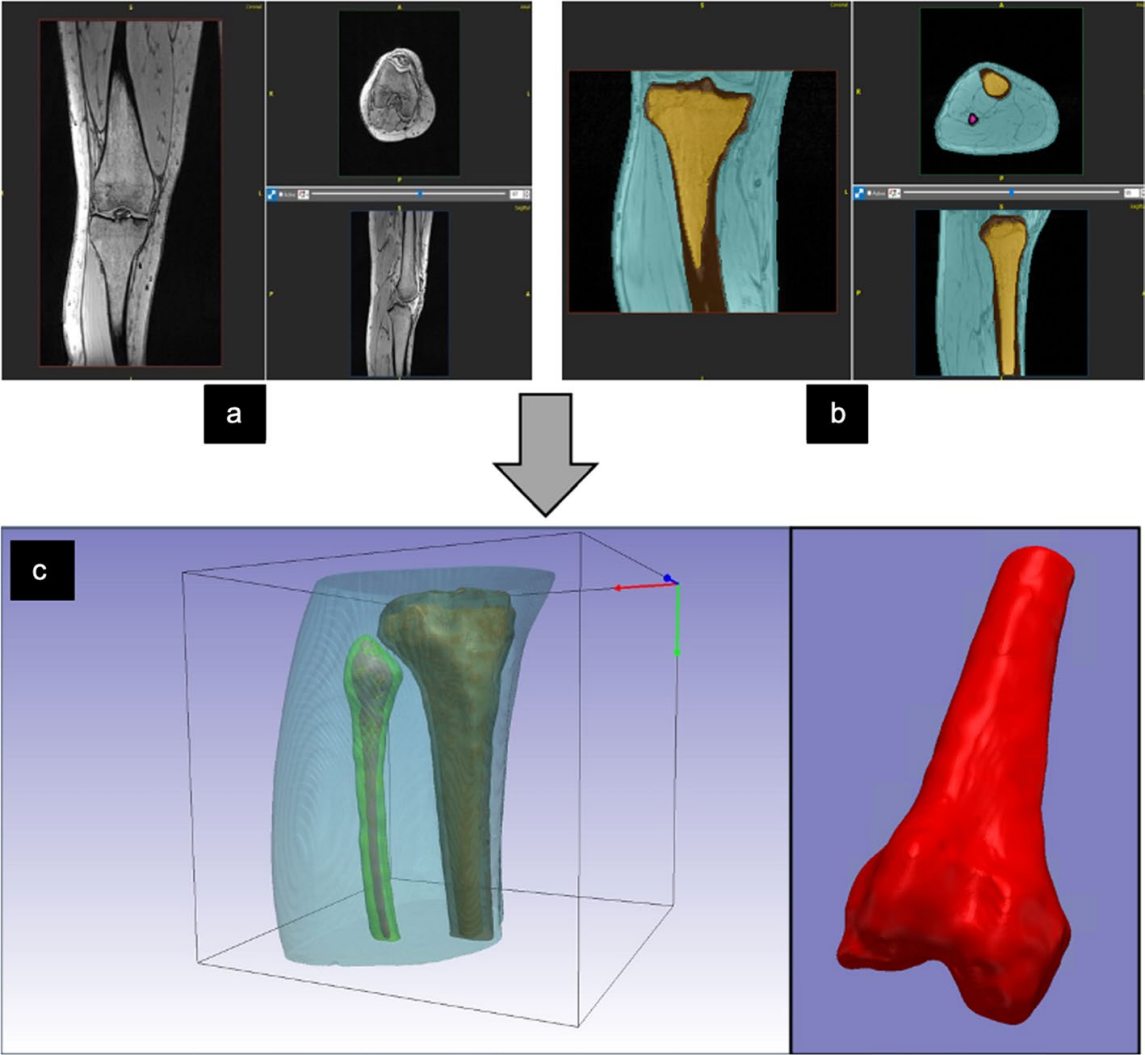
**a** Coronal, sagittal, and axial, view of knee MRI scan of one of the participants. **b** Segmented cortical and trabecular bone of knee joint MRI Images. **c** the exported 3D STL model of the segmented cortical, trabecular bone of the knee joint

The cortical and trabecular bones were segmented from the MRI DICOM images of the tibia and femur using ScanIP software (Synopsis Simpleware, Ver. 2020, Sunnyvale US) to create a 3D knee surface model consisting of cortical and trabecular compartments in every subject scan (Fig. 1). A threshold for image pixel intensity values was used to segment each compartment automatically which was then edited manually to delete any pixels segmented outside of the compartments. The surface models were then exported into stereolithography STL file format.

**Fig. 2:**
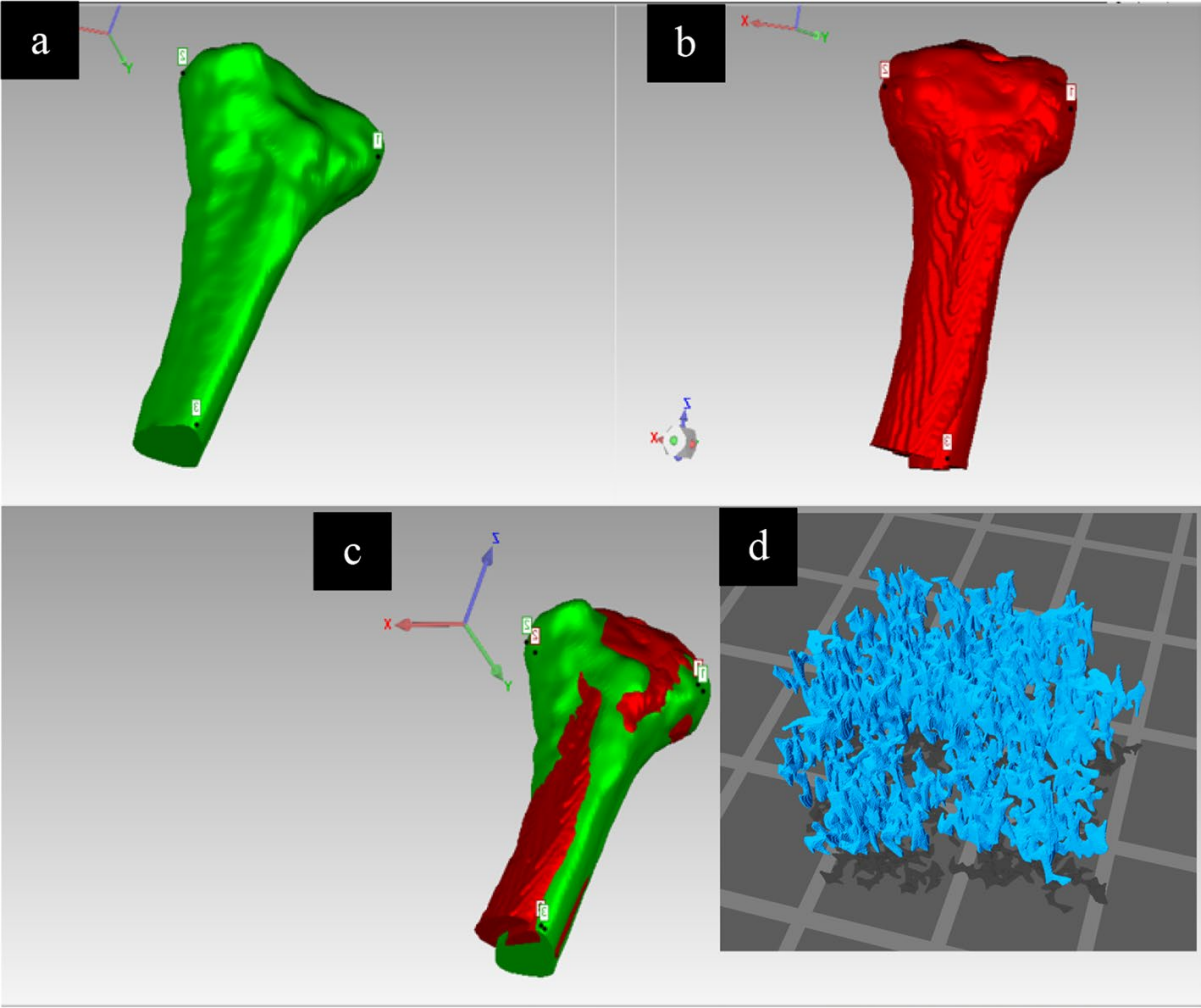
Manual Alignment of the 5 male tibia models. **a** the reference model. **b** is one of the models being aligned. **c** the result of the alignment of all the tibia models. **d** 3D model segmented from trabecular bone structures

One average 3D shape and volume model of the tibia and femur was based on the five male models and the other one from the five female models was created using (Geomagic Wrap, v.2021; 3D Systems) which is a software capable of editing and creating 3D surface models. The average tibia model was created by loading the five male tibia surface models into the software and aligning them manually using the global registration and alignment functions. One of the male tibia models was fixed and used as a reference while the rest of the models were aligned using multiple registration points applied to several locations in each model (Fig. 2). After the alignment and registration of the five models, the averaging function in the software was used to calculate the geometry and the volume of the models and create a new average volume TKR model with conserved average geometry and distinct cortical and trabecular bone compartments. The process of the alignment, registration, and models averaging was repeated for the male femur and female tibia and femur models. 3D slicer Chitubox software [17] was used to apply inverse modelling to the cortical and trabecular compartments of the average male and female phantoms to turn the solid models into hollow ones with distinct hollow compartments. This was done using the hollow tool in the software to create compartments with a wall thickness of 1 mm and hollowing precision of 80% to make the internal walls smooth. This inverse modelling is to allow for injecting fluids into the model's compartments. Finally, a trabeculae model segmented from one participant's MRI scan was inserted inside the empty trabecular compartment of the average male and female TKR models to create porous trabecular structures mimicking human trabecular bone (Fig. 2). ScanIP and Geomagic Wrap were used for generating and editing the TKR model because as they were already available in the lab and the authors had prior knowledge of their use.

### Modelling implant insertion

**Fig. 3:**
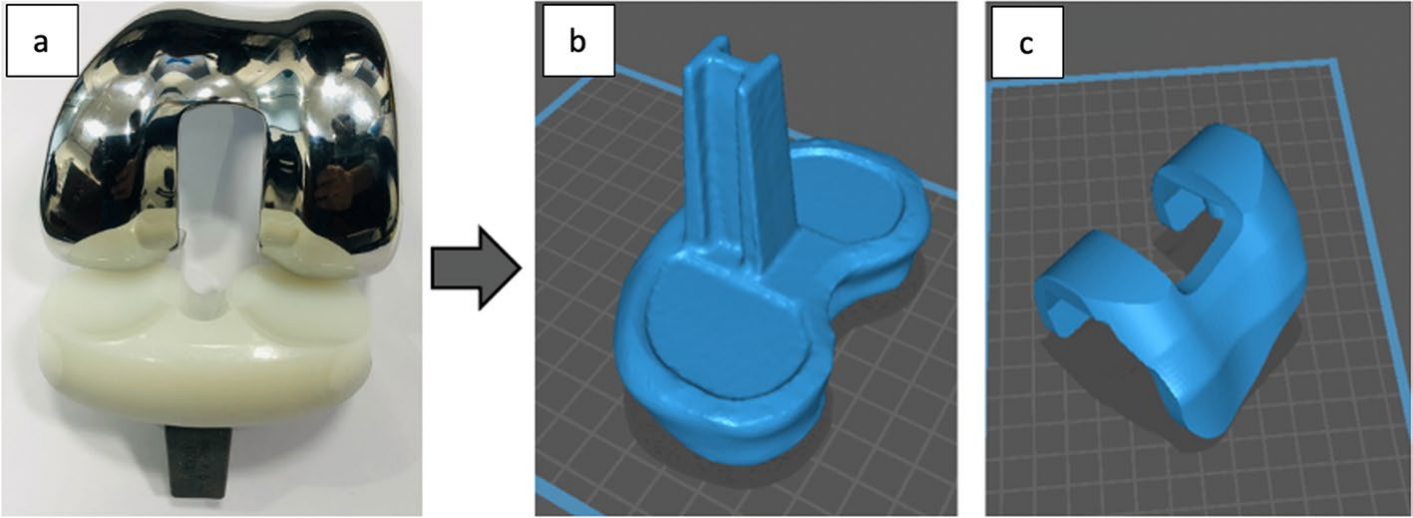
**a** Physical femur and tibia prostheses. **b** a 3D surface model of the tibial prosthesis metal compartment. **c** a 3D surface model of the femur prosthesis metal compartment

A 3D model of the tibial TKR prosthesis was created by scanning it using Polycam software with an iOS phone camera that employs light detection and ranging technology (LiDAR) [18] to create 3D models. This method is applied by continuous image capturing while rotating the prosthesis to cover it from multiple angles and reconstructing the images to create a 3D image. The 3D scan was performed by placing the prosthesis on a rotator and fixing the camera at a distance of 10 cm in a well-lit room. The prosthesis was then flipped and rotated again to cover its upper and lower sides. The tibia metal compartment model was created successfully with a similar shape to the real one with high accuracy but with a smaller size. This model was later edited, smoothed, and scaled to match the dimensions of the physical one using Geomagic wrap. However, capturing the femur metal compartment was not successful, probably due to the high irregularity of its shape which causes images to overlap at some angles and corrupt the final 3D reconstruction. To solve this problem, the geometries of the femur TKR prostheses were measured and drawn manually using SolidWorks software to create a 3D model. These CAD models were then used for the virtual TKR surgery, in which it was inserted into the average knee models, creating the insertion site by Boolean operations. This approach created a void volume for the physical insertion of the knee prosthesis which would be used in the phantom scan (Fig. 3).

The final step was to undertake virtual TKR surgery on the average male & female 3D models using the Chitubox software to merge the metal prostheses with the anatomical models. The tibia prostheses model was aligned and subtracted from the proximal part of the average male and female tibia models. After that, the void model of the tibia prostheses was merged with the average male and female tibia models to create an integrated tibia model with a metal prosthesis slot. The same process was applied to the femur compartment to create an integrated femur model with a void metal prosthesis slot. Before printing the models, a 3 mm hole was created on the lower side of each model compartment to facilitate cleaning and later fluid injection.

### 3D printing of TKR phantoms

The 3D surface models of the male and female TKR phantoms with an empty cortical compartment and porous trabecular compartment were printed with a 3D photopolymer resin printer (Elegoo Saturn) using Acrylonitrile butadiene styrene-like photopolymer resin (ELEGOO ABS-like) which is light-sensitive and commonly used for high accuracy 3D printing. The printing settings were selected based on the resin manufacturer’s recommended settings except bottom exposure time and bottom exposure time which were modified based on the results of several test prints carried out using a resin calibration tester STL model downloaded from the Frozen 3D website [19] to ensure the optimum printing resolution (Table 2). The phantoms were printed on a build angle of 180 degrees starting from the distal part of the tibia and proximal part of the femur model to reduce the number of supporting struts needed during the printing. Subsequently, the printed models were injected and washed with 99.9% pure isopropyl Alcohol to dissolve the remaining resin from the inside and outside walls. The printed models were then put in a strip-curing ultraviolet light system (ANYCUBIC Wash and Cure Plus) for 20 min to cure the remaining resin on the model's walls, make them stronger, and close any potential micro holes that might happen during phantom modelling or printing.

**Table 2:** 3D printer settings

Layer height	0.05 mm	Bottom lift distance	7 mm
Bottom layer count	5	Lifting distance	7 mm
Exposure time	3 s	Bottom retract distance	7 mm
Bottom exposure time	60 s	Retract distance	7 mm
Transition layer count	10	Bottom lift speed	70 mm/min
Transition type	Linear	Lifting speed	70 mm/min
Transition time decrement	2.5 s	Bottom retract speed	210 mm/min
Rest time after retract	0.5 s	Retract speed	210 mm/min

### Modelling X-ray attenuation and radioactive tracer distribution

**Fig. 4:**
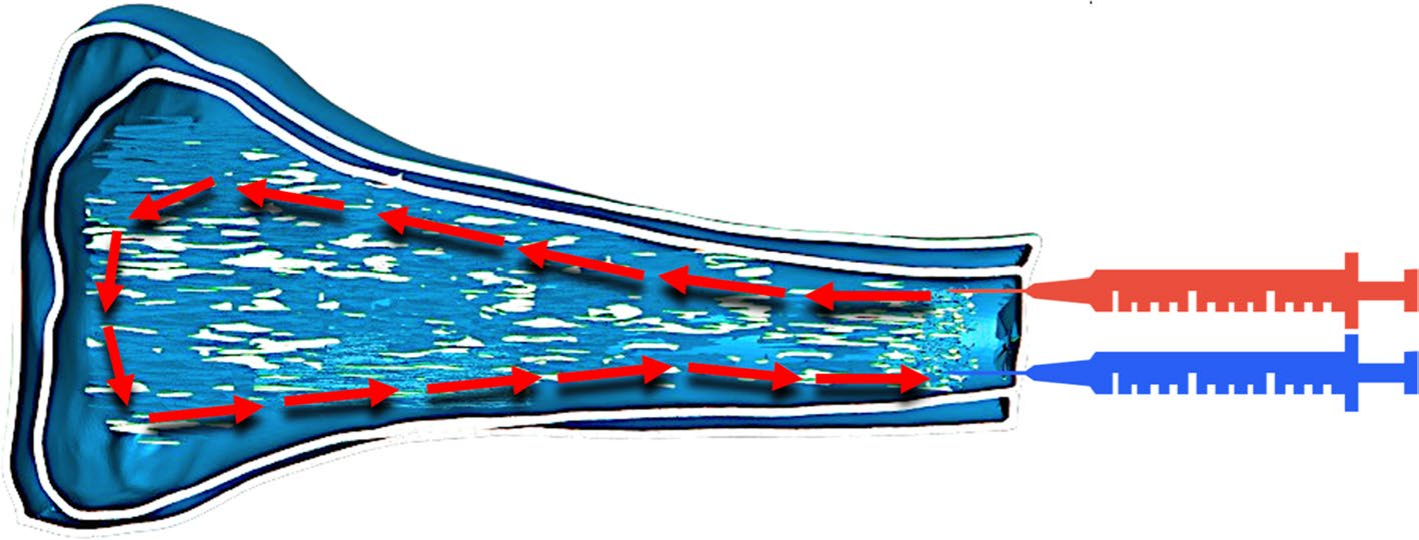
Schematic diagram showing the method of activity injection into the trabecular bone compartment of the phantom. The inlet syringe contains the FDG injected (red), and the outlet syringe (blue) is partially filled with saline to receive the excess saline coming back from the phantom during the injection (red arrows)

Normal saline and iodinated contrast media (Omnipaque 350 mg I/mL; GE Healthcare, USA) were used to simulate the trabecular and cortical bone attenuation of the x-ray and ^18^F-FDG was used to simulate the ^18^F-NaF uptake and distribution in trabecular bone due to its low cost and availability. Tap water was injected into phantom compartments before any simulation experiment to test them for any possible leaks. To ensure safe injection during phantom handling, one inlet and one output cannula were inserted into the trabecular compartment and sealed with resin to facilitate injection and avoid radioactivity leaks. The inlet cannula was connected to the radiotracer syringe. The outlet cannula was attached to a bleeding syringe containing normal saline to equalise pressure inside the phantom during the injection and make an air-free closed injection system (Fig. 4).

After preliminary CT scans using 120 kV and 35 mA of several vials filled with different concentrations of iodinated contrast media and saline, the cortical compartment of the phantoms was injected with 20% iodinated contrast media diluted with 80% of normal saline which was found to be the best concentration to mimic the cortical bone HU range of + 300 to + 1000 reported in the literature [20, 21]. The porous trabecular compartment was injected with normal saline so both the resin and saline mimic the trabecular HU range of 50–300 as reported in the literature [20]. To assess the phantom with PET/CT, the trabecular compartments of the male and the female TKR phantoms were injected with 5 MBq of FDG. This amount of activity is arbitrary just to test the phantom concept for PET/CT.

The TKR phantoms were scanned with a Siemens PET/CT scanner Biograph128_Vision 600 Edge (Siemens Healthineers, Germany) in static scan mode for 10 min with one-bed position using 120 kV, 35 mA for the CT scan and 3 mm slice thickness for PET/CT images. Reconstructions were performed with 8 iterations, and 5 subsets were used to reconstruct the PET/CT images.

**Fig. 5:**
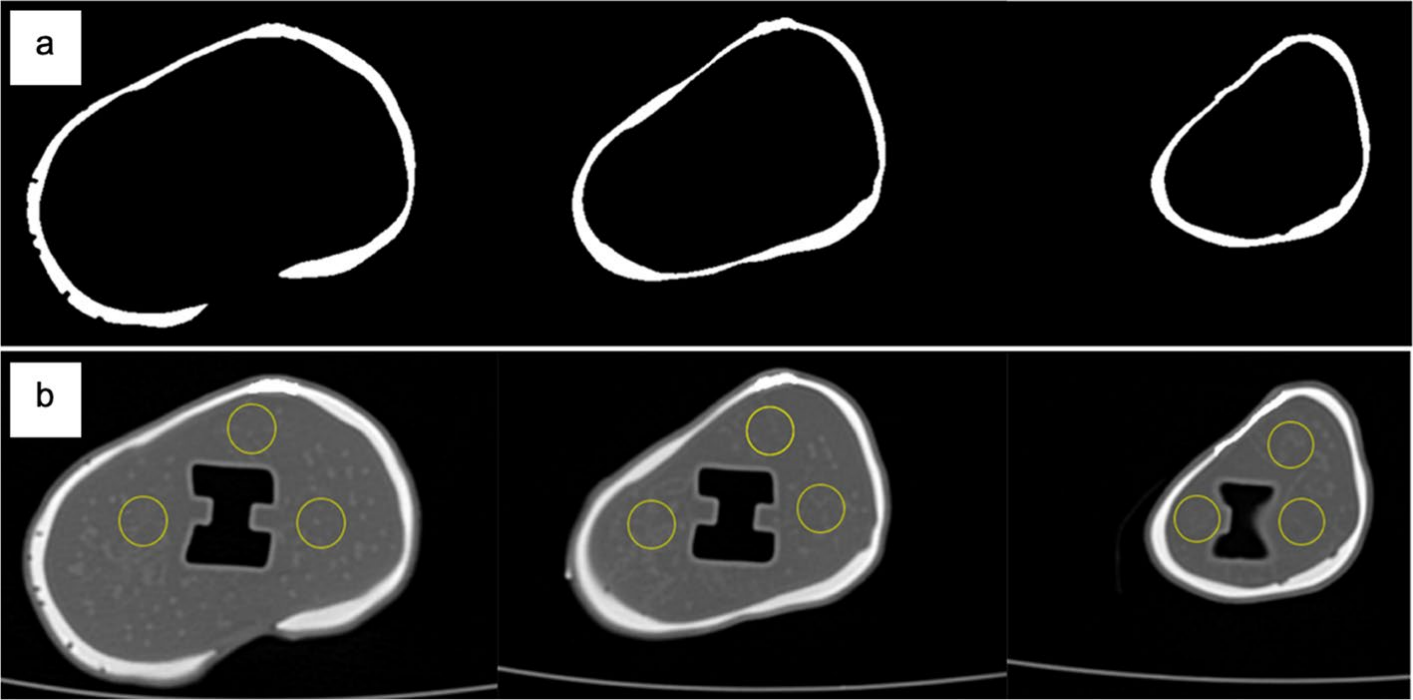
**a** left, middle, and right images show cortical bone compartment segmented from the top, middle, and bottom slices around the tibial prosthetic insert to calculate average HU. **b** shows the same slices used to calculate the average trabecular HU obtained from 3 circular ROIs from the 3 slices

The whole cortical compartment from the bottom, middle and top (around the tibial prosthetic insert) of the femur and tibia were segmented from three axial CT slices of each phantom CT scan to measure their average HU and the standard deviation SD (Fig. 5a). The average trabecular HU and the SD were obtained from three regions of interest ROIs drawn on three CT slices from the bottom, middle and top (around the tibial prosthetic insert) of the femur and tibia of each phantom (Fig. 5b). In addition, a line profile was drawn across the axial PET/CT images of the male and female tibia and femur phantoms to plot their HU profiles and activity distribution profiles.

## Results

### Qualitative and quantitative results of the TKR phantom printing and CT modelling

**Fig. 6:**
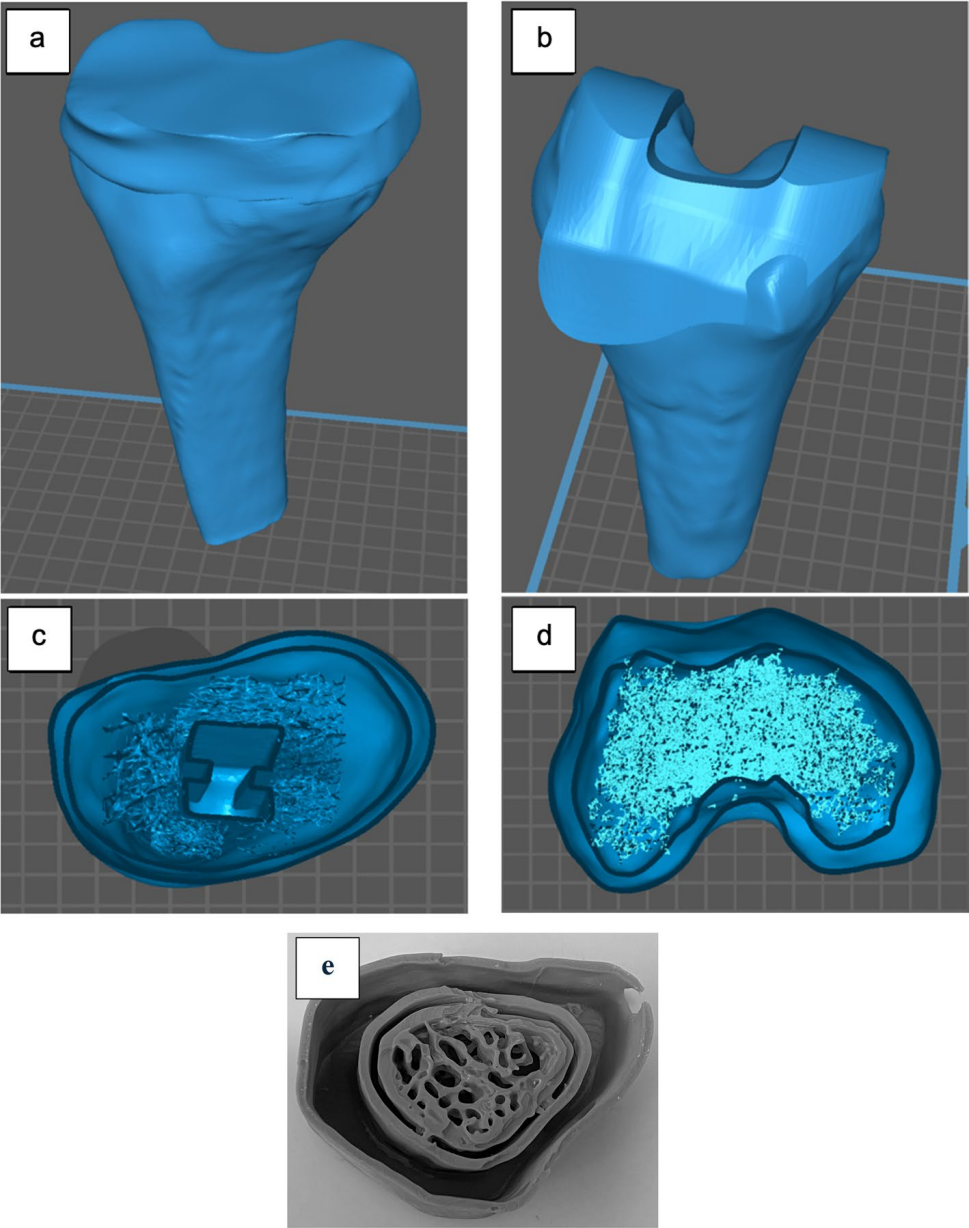
**a** and **b** show the tibia and femur of one of the TKR models respectively. **c** and **d** show a cross-section view of the tibia and femur TKR model showing the cortical and trabecular compartments. **e** shows a cross-section of the physical TKR phantom after printing

One 3D male and one female 3D TKR models with hollow injectable cortical and trabecular compartments were created successfully (Fig. 6). Both compartments could be injected with attenuating fluids and ^18^F-FDG or ^18^F-NaF to mimic radiological features, artefacts, and radioactivity biodistribution in human TKR PET/CT scans.

**Fig. 7:**
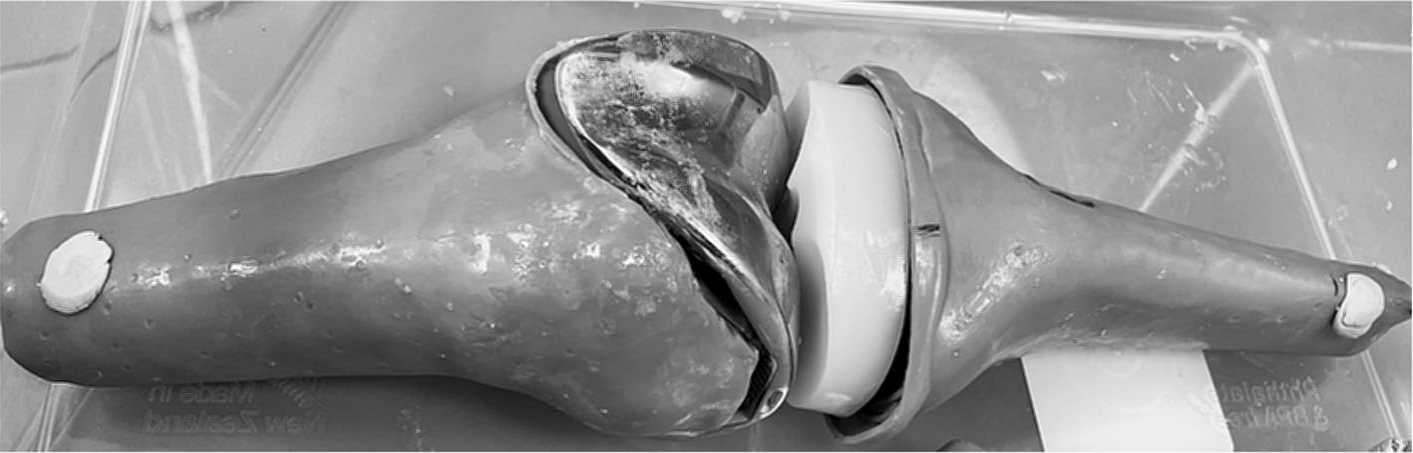
Example of one of the printed TKR phantom with metal prostheses insert

**Fig. 8:**
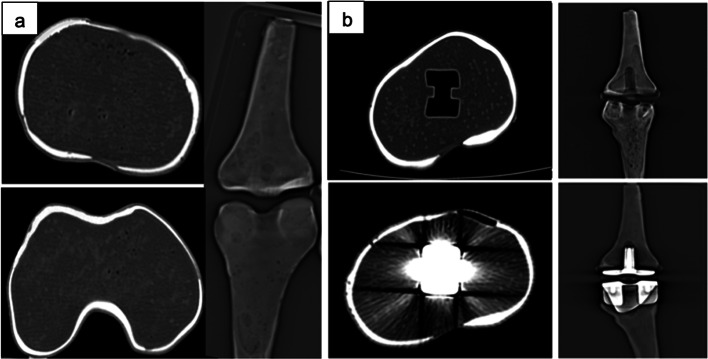
**a** Show axial view and tomogram of the female tibia and femur phantoms before the virtual TKR surgery of the model. **b** Top row images show a tomogram and axial view of the female model after virtual TKR surgery without knee prosthesis. Bottom row shows a tomogram and axial view of the female model with knee prosthesis

The final male and female TKR models were printed separately successfully using a 3D photopolymer resin printer (Fig. 7). The average printing time for the male and female phantoms was 9 h and 25 min each. No defects or leaks between the compartments were presented either visually or in the CT scans. The height, width, and depth of the printed phantoms were (35 × 11 × 6.4) cm for the male TKR and (36 × 11 × 6) cm for the female TKR. Phantoms CT scans showed the cortical and trabecular compartments to have similar visual radiographic features to human ones with a similar pattern of metal artefacts and ground truth images with no artefact, with an example shown in Fig. 8.

**Fig. 9:**
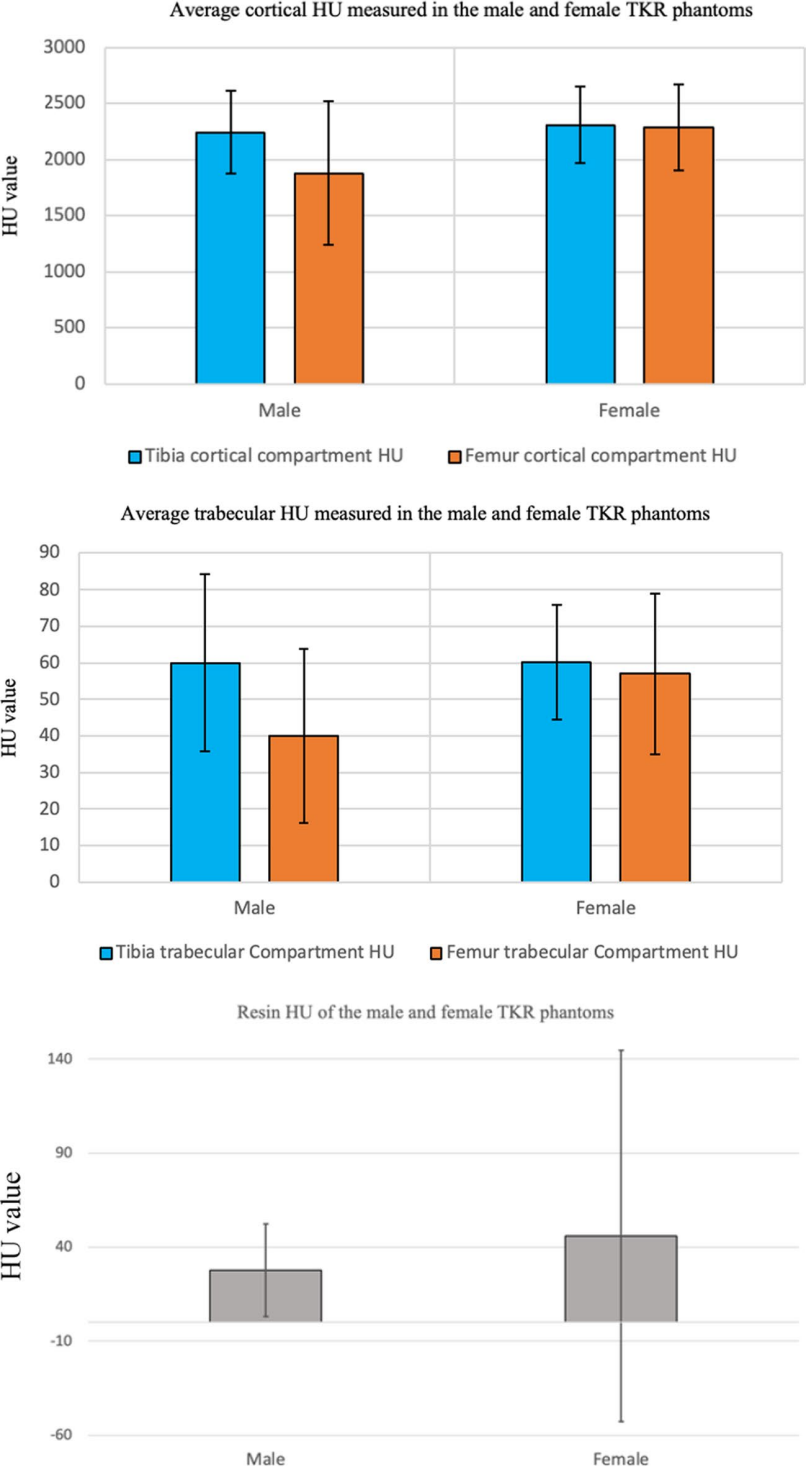
Bar charts show the mean HU value with standard deviation of the cortical, trabecular compartments and resin for male and female TKR phantoms

**Fig. 10:**
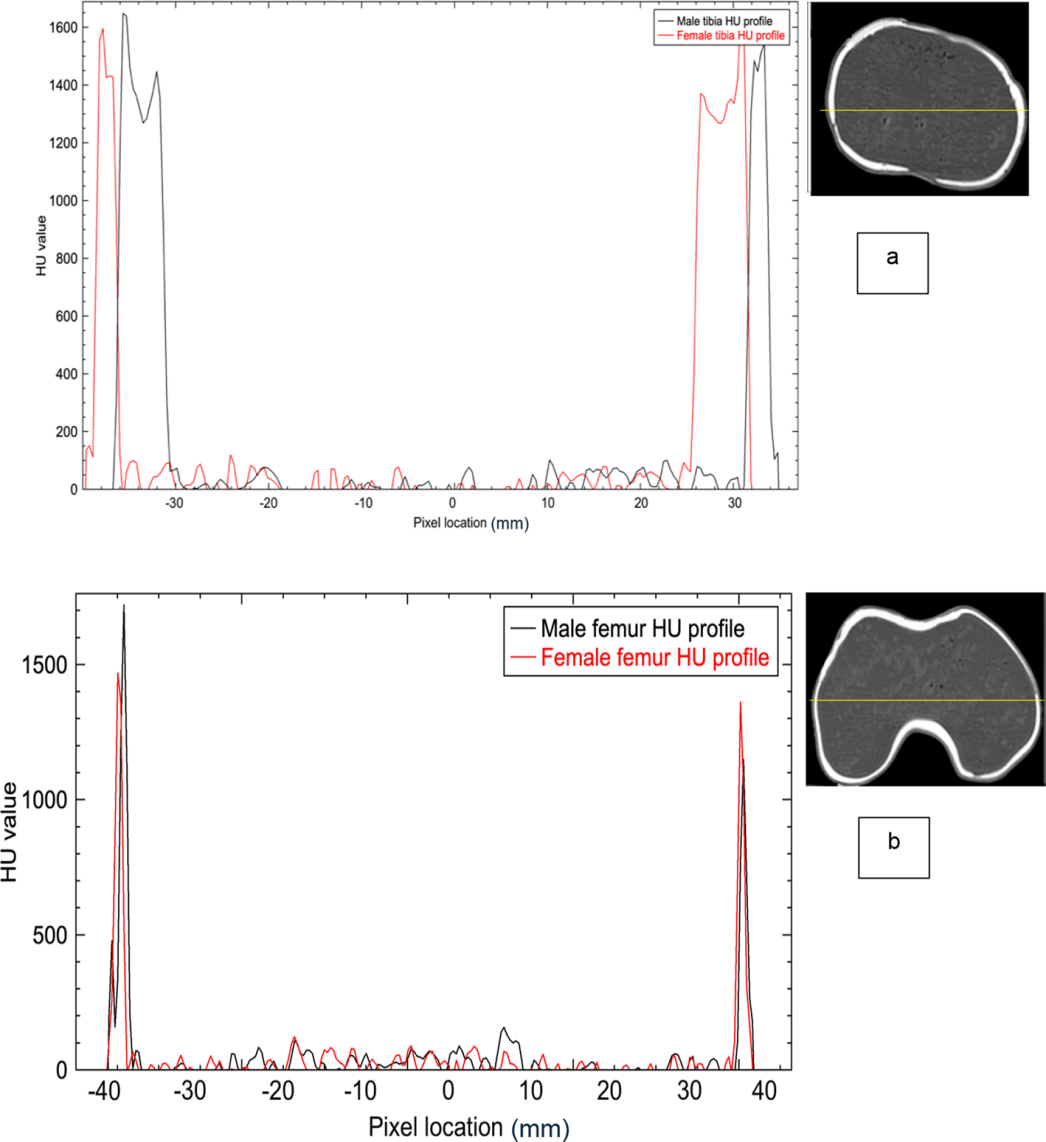
**a** HU profile plot of the cortical and trabecular compartments obtained from axial CT image of the proximal part male and female tibia phantom before virtual surgery. **b** HU profile plot of the cortical and trabecular compartments obtained from axial CT image of the distal part male and female femur phantom before virtual surgery

The average HU and standard deviation of the cortical, trabecular, and resin compartments measured from the female and male phantoms and their comparison to the clinically reported values are presented in Fig. 9. Figure 10 shows the HU profile obtained from a line drawn on the cortical and trabecular bone compartments of the male and female tibia and femur phantoms.

### Qualitative and quantitative results of the TKR phantom PET modelling

**Fig. 11:**
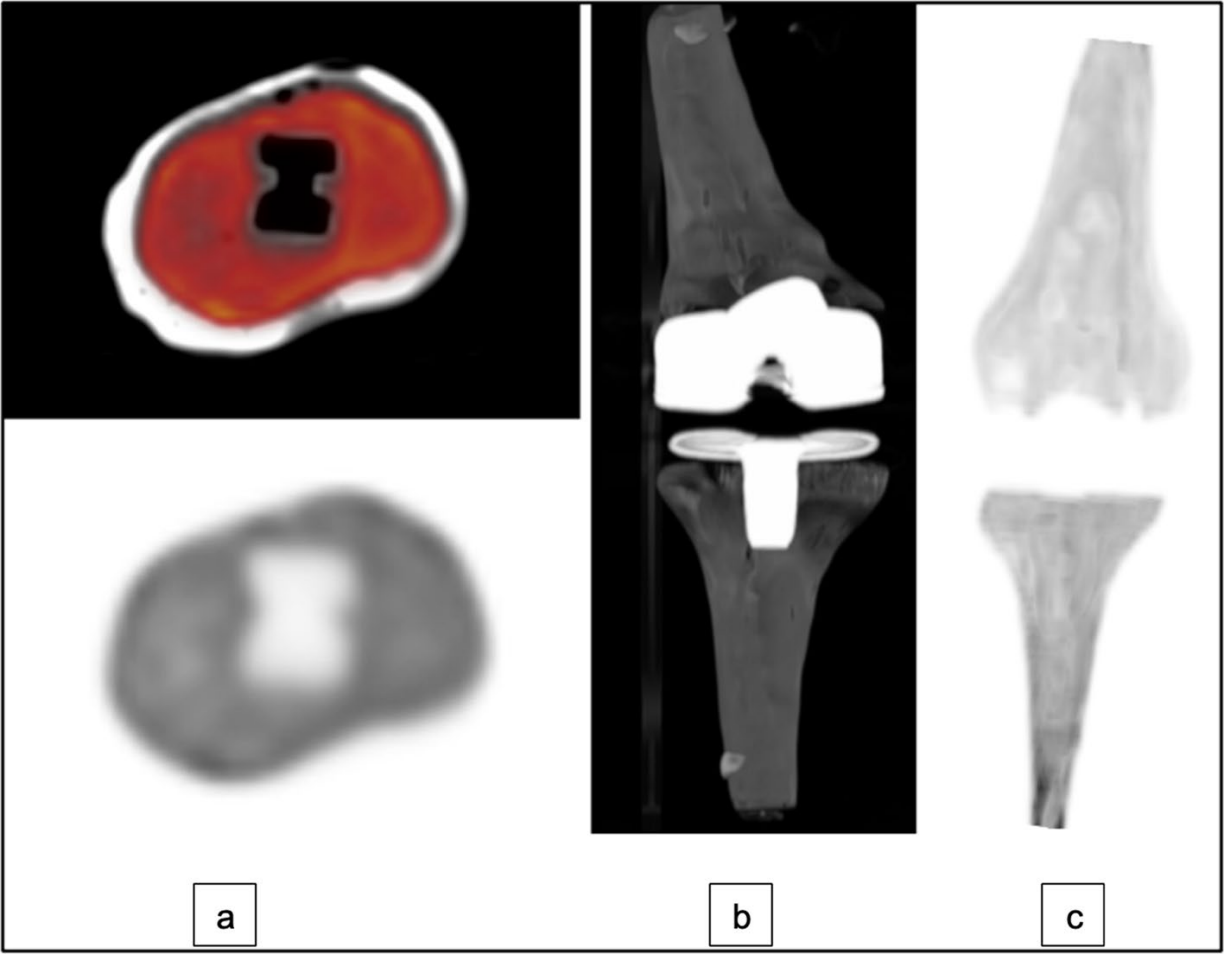
**a** show an example of axial fused PET/CT and PET scan from the male phantom without metal insert. **b** 3D view of the phantom CT scan with prostheses. **c** a 3D view of the FDG distribution in the phantom PET scan

**Fig. 12:**
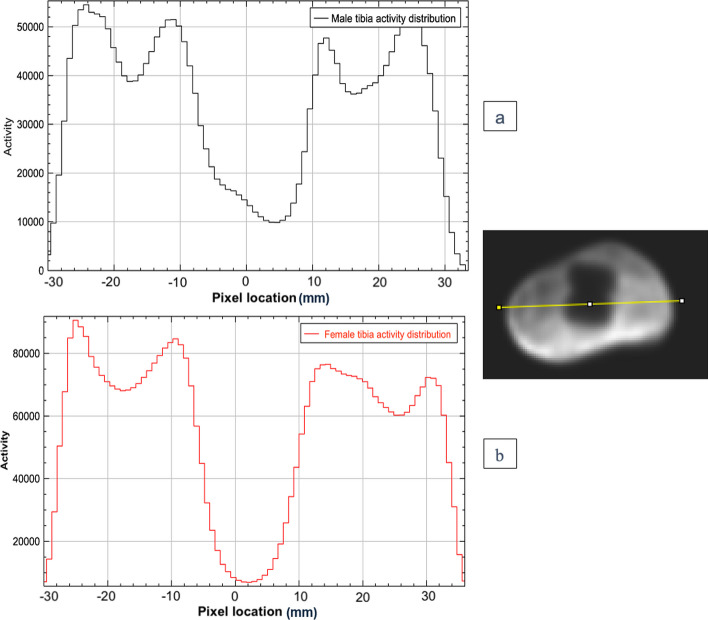
**a** Activity distribution profile around the prosthesis across the axial view of the proximal part of the male tibial phantom PET image showing approximately even distribution within the phantom. **b** Activity distribution around the prosthesis across the axial view of the proximal part of the female tibial phantom PET image showing approximately even distribution within the phantom

**Fig. 13:**
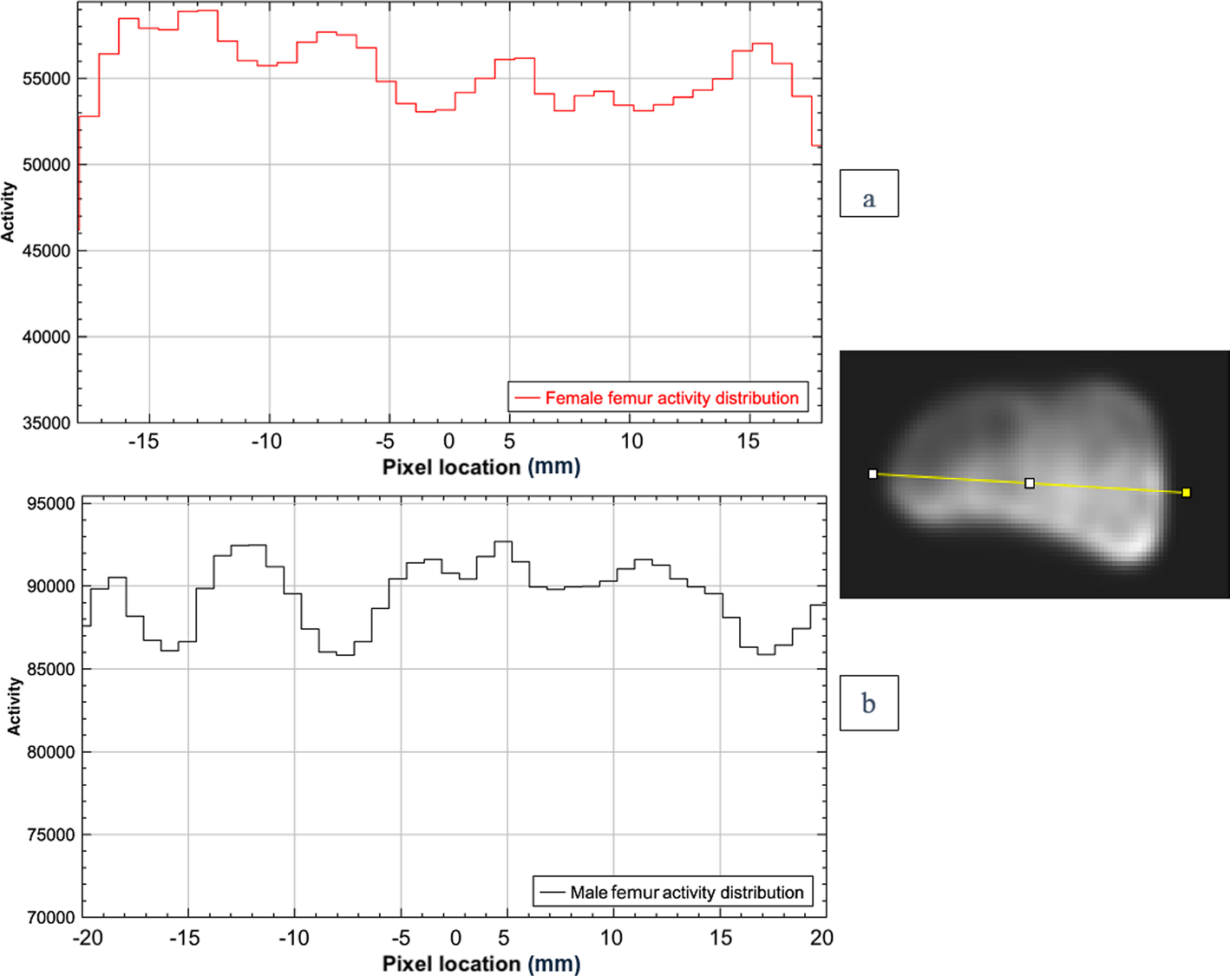
**a** Activity distribution across the axial view of the distal part of the female femur phantom PET image showing approximately even distribution within the phantom. **b** Activity distribution across the axial view of the distal part of the male femur phantom PET image showing approximately even distribution within the phantom

The phantoms were injected with FDG successfully and no leaks were observed either during or after injection. The PET/CT scan shows a good distribution homogeneity of the FDG within the trabecular compartments of the tibia and the femur phantoms as shown in Fig. 11. A line profile plot shows approximately an even activity distribution around prosthesis within the male and female tibial phantoms (Fig. 12). Likewise, Fig. 13 shows approximately an even activity distribution within the male and female femur phantoms.

## Discussion

This study presents a novel design and 3D printing of an anthropomorphic, population-based, and customisable male and female TKR phantom which can be filled with any bone attenuation equivalent fluids and injected with any appropriate radiopharmaceutical to mimic human TKR PET/CT scans. A range of solutions can be diluted in water and injected into the TKR phantom to simulate either cortical or trabecular bone such as iodinated contrast media or dipotassium hydrogen phosphate (K_2_HPO_4_) which has been used in previous studies to mimic a range of HU values [14, 22]. In our experiment, iodinated contrast media was used as it was easy to source and it allowed the TKR phantoms to be used with adjustable HU values for testing any optimisation method applied to reduce the associated metal artefacts that arise in the PET/CT scans. The fluidic domain, instead of solids used in common phantoms, enables the injection of different radioactive tracers such as ^18^F-FDG or ^18^F-NaF. The injected activity can be adjusted to either mimic the normal human bone uptake or to use the minimum activity required for a reliable measurement of the standardised uptake value SUV of ^18^F-FDG in phantom studies [23] to reduce radiation exposure to the operator and to ensure consistent (SUV) measurement across multiple PET/CT scans.

On the model design aspect of the TKR phantom, the model developed can be customised to reflect different bone abnormal cases including osteoporosis, osteoarthritis, and hypermineralisation by modifying the trabecular compartment porosity and changing the concentration of the injected attenuating fluid. Moreover, small injectable compartments can be embedded in the trabecular compartment around the knee prosthesis to simulate bone malignancies which helps test the performance of MAR methods in improving the visibility and the quantification of hot spots around the prosthesis. As the model design of the phantom is population-based, it allows for representing the overall variation of the geometrical and radiological features between human subjects.

On the x-ray attenuation aspects of our phantom, the approach presented HU values for the cortical and the trabecular compartments similar to that reported in humans [20, 24]. However, the femur trabecular compartment of the male phantom was 40 (23.8 SD), slightly lower than the 50 suggested in the literature. This may be due to the high porosity of the femur trabecular compartment. This issue can be resolved by injecting a small amount of the iodine inside the trabecular compartment as required to get the appropriate HU value. Overall, our phantom has shown similar levels of x-ray attenuation features to previous studies [25] using solid materials only, including wall filler and polystyrene, to house a metal implant for TKR and to assess metal artefact reduction (MAR) methods.

Our phantom design has introduced a different level of functionalities by enabling the assessments in both PET and CT images with the fluid-based material. The images produced by Tins et al. CT phantom can be assessed visually and physical parameters such as signal-to-noise ratio can be measured [26] but it cannot be used to assess the overestimation and underestimation of image quality caused by the applied MAR methods, as the phantom cannot be used to produce ground truth images which have no metal artefacts present. In contrast, our phantoms can be scanned with and without knee prosthesis inserts to give scans with metal artefacts that can be judged against a reference PET/CT scan with no metal artefact, allowing accurate comparisons to be made between the applied MAR methods.

In two studies by Van der Vos et al. [27] and Kennedy et al. [28], a simple phantom consisting of hip metallic implants immersed in a solution of ^18^F-FDG and water was used to assess the performance of MAR algorithms used to correct CT image metal artefacts applied to the corresponding PET images. Although these phantoms could be used for visual and some physical assessment of the PET/CT scans, they do not simulate human tissue structures surrounding the prosthesis which limits the use of physical metrics related to the CT scans used for PET image correction. As the hip metal phantom cannot be used to generate ground truth scans and the prosthesis is surrounded by only water physical assessment parameters such as structural similarity index (SSIM), normalized root mean square difference (NRMSD), mean relative error (MRE) and mean absolute deviation (MAD) between the corrected and ground truth images cannot be generated [29–31].

Andersson et al. [32] used a calf hip bone to assess different commercial MAR techniques to improve hip prosthesis CT images. Although using animal hips could give radiological features close to human ones, it does not provide accurate shape and geometry representative of humans. Moreover, cadaver phantoms cannot be injected with radiopharmaceuticals and cannot be stored for a long time which makes them unsuitable for PET/CT scans and repeated scans over an extended timescale.

The main limitation of the phantom is that it can be only used with the type of TKR used in the phantom design. If another type of TKR is to be examined, then another phantom print using a 3D model of the required TKR geometry must be generated and merged with the knee joint model. As the prosthesis models were created either by manual or by reversal modelling, a small variation between the physical prosthesis and their 3D model’s geometry is expected due to the inaccuracy associated with this kind of modelling process [33]. The phantom cannot be handled remotely and the exposure of the operator to the radiation activity is unavoidable, but exposure can be reduced by following the principles of radiation safety [34]. The authors have used expensive softwares for generating and processing the TKR model as they are already available in the local lab and the authors have prior knowledge of how to use them. However, other alternative free softwares such as 3D slicer can be used to reduce the cost of phantom fabrication.

## Conclusion

In conclusion, this study shows the process of segmenting, modelling, printing, and validation of 3D-printed TKR phantoms that simulate the geometry, shape, and radiological features of human PET/CT TKR. This demonstrates that 3D printing is a valuable method in the fabrication of nuclear medicine and PET/CT phantoms that can be injected with a range of attenuating solutions and radiopharmaceuticals. Future work could focus on adding varying-sized lesion-like compartments within the trabecular bone compartment around the metal implant. This would allow for testing the performance of MAR methods in the detection of periprosthetic lesions.

## Data Availability

The datasets generated during and/or analysed during the current study are available from the corresponding author on reasonable request.
